# Robotic bariatric surgery reduces morbidity for revisional gastric bypass when compared to laparoscopic: outcome of 8-year MBSAQIP analysis of over 40,000 cases

**DOI:** 10.1007/s00464-024-11192-0

**Published:** 2024-08-23

**Authors:** Graham J. Spurzem, Ryan C. Broderick, Emily K. Kunkel, Hannah M. Hollandsworth, Bryan J. Sandler, Garth R. Jacobsen, Santiago Horgan

**Affiliations:** Division of Minimally Invasive Surgery, Department of Surgery, University of California San Diego, 9300 Campus Point Dr La Jolla, San Diego, CA 92037 USA

**Keywords:** Revisional bariatric surgery, Robotic surgery, MBSAQIP, Sleeve gastrectomy, Roux-en-Y gastric bypass

## Abstract

**Introduction:**

Robotic-assisted metabolic and bariatric surgery (MBS) is gaining popularity. Revisional MBS is associated with higher perioperative morbidity compared to primary MBS. The optimal surgical approach to minimize complications in these complex cases is unclear. The goal of this study was to assess robot utilization in revisional MBS and compare laparoscopic and robotic revisional MBS outcomes in the Metabolic and Bariatric Surgery Accreditation and Quality Improvement Program (MBSAQIP) database.

**Methods:**

A retrospective review of the MBSAQIP database was performed identifying revisional sleeve gastrectomy (SG) and Roux-en-Y gastric bypass (RYGB) cases from 2015 to 2022. Primary MBS, open/emergent cases, cases converted to another approach, and combined cases other than esophagogastroduodenoscopy were excluded. 30-Day outcomes for laparoscopic and robotic cases were compared using multivariate logistic regression adjusting for patient demographics, comorbidities, and operative variables.

**Results:**

41,404 Cases (14,474 SG; 26,930 RYGB) were identified. From 2015 to 2022, the percentage of revisional SG and RYGB cases performed robotically increased from 6.1% and 7.3% to 24.2% and 32.0% respectively. Laparoscopic SG had similar rates of overall morbidity, leak, bleeding, readmission, reoperation, and length of stay compared to robotic. Laparoscopic RYGB had significantly higher rates of overall morbidity (6.2% vs. 4.8%, *p* < 0.001, AOR 0.80 [0.70–0.93]), blood transfusion (1.5% vs. 1.0%, *p* < 0.05, AOR 0.74 [0.55–0.99]), superficial incisional SSI (1.2% vs. 0.4%, *p* < 0.001, AOR 0.30 [0.19–0.47]), and longer length of stay (1.87 vs. 1.76 days, *p* < 0.001) compared to robotic. Laparoscopic operative times were significantly shorter than robotic (SG: 86.4 ± 45.8 vs. 113.5 ± 51.7 min; RYGB: 130.7 ± 64.7 vs. 165.5 ± 66.8 min, *p* < 0.001).

**Conclusion:**

Robot utilization in revisional bariatric surgery is increasing. Robotic surgery has lower postoperative morbidity and shorter length of stay in revisional RYGB when compared to laparoscopic. Robotic platforms may have the capacity to improve the delivery of care for patients undergoing revisional bariatric surgery.

Metabolic and bariatric surgery (MBS) has been established as the most effective treatment for obesity [1, 2]. MBS is gaining popularity as a result, with sleeve gastrectomy (SG) and Roux-en-Y gastric bypass (RYGB) being the most common procedures [3]. However, primary MBS is often complicated by weight regain, postoperative reflux, metabolic disorders, and other anatomic complications that can require a revision or conversion operation. For example, an estimated 10–20% of patients experience weight regain after primary MBS or fail to achieve significant weight loss altogether [4–6]. Revisional cases are the third most common type of MBS in the United States (US), with over 30,000 revisions performed each year on average since 2016 [3].

Revisional MBS can be technically challenging due to the presence of altered anatomy in reoperative fields with adhesions [7, 8]. Consequently, revisional MBS is generally associated with higher morbidity and longer operative times compared to primary MBS [5, 9–11]. The rise of robotic surgical platforms, with their improved dexterity and visualization, has led many to apply this new technology to revisional MBS in an effort to overcome the shortcomings of conventional laparoscopy [12–14]. The optimal surgical approach for these complex cases, however, remains unclear. Several case series, national database studies, and meta-analyses comparing laparoscopic and robotic revisional MBS have been performed with varying results [15–22].

The Metabolic and Bariatric Surgery Accreditation and Quality Improvement Program Participant Use Data File (MBSAQIP PUF) is the largest, bariatric-specific, clinical dataset in the US. Prior studies of the MBSAQIP PUF comparing laparoscopic and robotic revisional MBS have been performed using data through 2020 [20]. The MBSAQIP has since been updated through 2022 with the addition of multiple new variables. To our knowledge, there have been no studies comparing the outcomes of laparoscopic and robotic revisional MBS with these new data. The goal of this study was to perform the largest retrospective analysis of the MBSAQIP database comparing perioperative outcomes between laparoscopic and robotic-assisted revisional SG and RYGB cases. We also sought to assess robot utilization in minimally invasive revisional MBS over the 8-year period of available MBSAQIP data.

## Methods

### Study design and data source

A retrospective analysis of the 2015–2022 MBSAQIP PUF was performed identifying revisional SG and RYGB cases performed laparoscopically and robotically. The MBSAQIP is a database created by the American College of Surgeons (ACS) and American Society of Metabolic and Bariatric Surgery (ASMBS). All nationally accredited metabolic and bariatric surgery centers in the United States report outcomes to the MBSAQIP. All outcomes recorded in the dataset are 30-day outcomes. Data from each center are collected by trained clinical reviewers and audited. The MBSAQIP is a deidentified database, and this study was therefore exempt from institutional review board approval. The ACS, MBSAQIP, and the centers participating in the MBSAQIP are the source of the data used herein; they have not verified and are not responsible for the statistical validity of the data analysis or the conclusions derived by the authors.

### Case selection

**Fig. 1:**
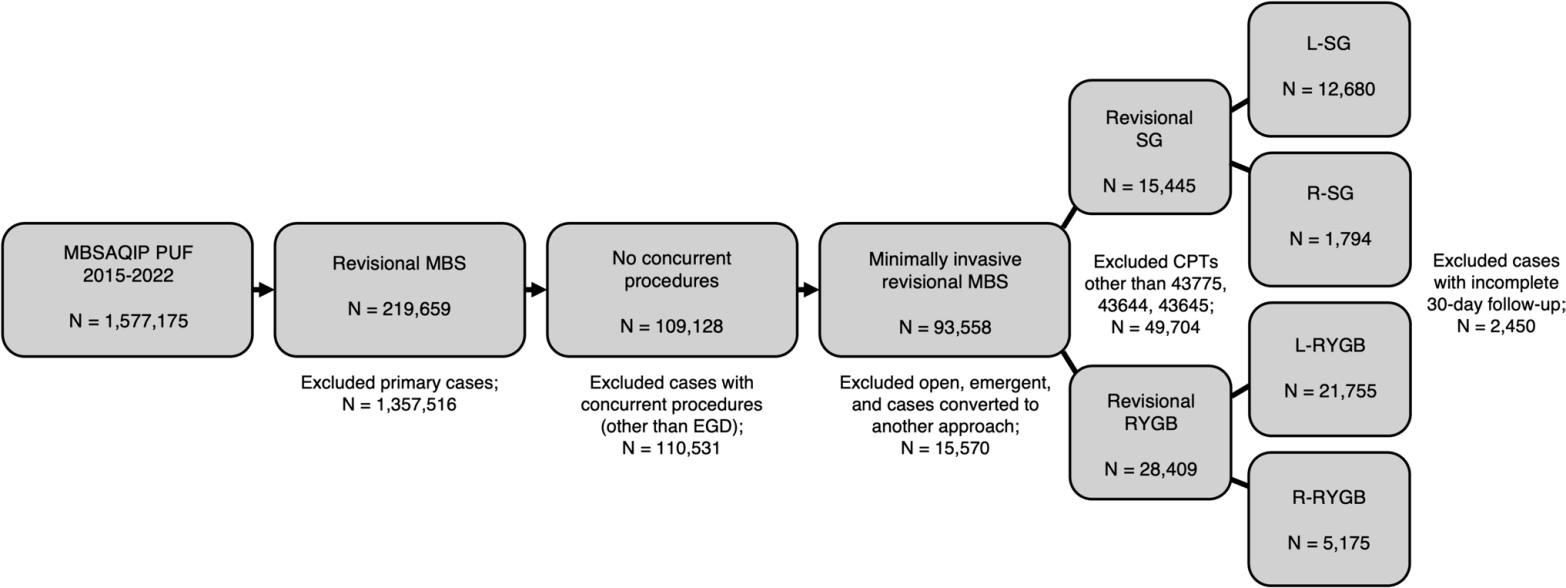
Case selection algorithm identifying revisional laparoscopic (L-) and robotic (R-) sleeve gastrectomy (SG) and Roux-en-Y gastric bypass (RYGB) cases from the MBSAQIP database. *MBSAQIP PUF*  Metabolic and Bariatric Surgery Accreditation and Quality Improvement Program Participant Use Data File, *EGD*  esophagogastroduodenoscopy

The case selection algorithm for this study is shown in Fig. 1. Revision and conversion MBS cases were first selected by excluding all other case types. Cases involving concurrent procedures other than esophagogastroduodenoscopy (EGD) were then removed. All surgical approaches other than laparoscopic and robotic were excluded. Cases converted to another surgical approach and emergent cases were also removed. SG and RYGB cases were then selected by current procedural terminology (CPT) codes 43775, 43644, and 43645. Standard laparoscopic and robotic-assisted cases were separated. Finally, cases with incomplete 30-day follow-up and missing data were excluded.

### Patient demographics and operative data

Patient demographic data included age, sex, American Society of Anesthesiologists (ASA) classification, and body mass index (BMI) closest to surgery. Comorbidities included hypertension, hyperlipidemia, dependent functional health status, current smoker within one year, diabetes mellitus, chronic steroid/immunosuppression use, chronic obstructive pulmonary disease (COPD), pulmonary embolism (PE), deep vein thrombosis (DVT) requiring therapy, preoperative therapeutic anticoagulation, inferior vena cava (IVC) filter, obstructive sleep apnea (OSA), gastroesophageal reflux disease (GERD), history of myocardial infarction (MI), previous cardiac surgery, renal insufficiency, and dialysis. Operative variables included leak test, drain placed, and operative time.

### Outcomes

30-Day outcomes included overall morbidity, anastomotic leak, bleeding, blood transfusion within 72 h of surgery, readmission, reoperation, reintervention, mortality, superficial incisional surgical site infection (SSI), deep incisional SSI, organ/space SSI, wound disruption, sepsis, septic shock, urinary tract infection (UTI), ventilator > 48 h, unplanned intubation, postoperative pneumonia, venous thrombosis requiring therapy, PE, stroke, unplanned admission to the intensive care unit (ICU), acute renal failure requiring dialysis, progressive renal insufficiency, cardiac arrest requiring cardiopulmonary resuscitation (CPR), myocardial infarction, and hospital length of stay. Robotic utilization as a percentage of total minimally invasive revisional MBS over the 8-year period was also analyzed.

From 2015 to 2019, there are no dedicated variables for anastomotic leak or bleeding. Beginning in 2020, the variables “Anastomotic/Staple Line Leak” (defined as a leak of endoluminal contents through an anastomosis or staple line from the MBS procedure) and “Gastrointestinal Tract Bleeding” (defined as bleeding of any portion of the gastrointestinal tract, which may be at a staple line, anastomosis, enterotomy, or ulcer) were included. We defined anastomotic leak for 2015–2019 as an aggregate of reoperation, readmission, and reintervention for suspected leak, drain present at 30 days, and organ/space SSI. For 2020–2022, leak was defined as an aggregate of reoperation, readmission, and reintervention for leak and the variable “Anastomotic/Staple Line Leak.” Bleeding for 2015–2019 was defined as an aggregate of reoperation, readmission, and reintervention for suspected bleeding. For 2020–2022, an aggregate of reoperation, readmission, and reintervention for gastrointestinal tract bleeding and the variable “Gastrointestinal Tract Bleeding” was used. This aggregate complication methodology for 2015–2019 data was previously described by Berger et al. [23].

Overall morbidity was defined as an aggregate of anastomotic leak, bleeding, blood transfusion within 72 h of surgery, superficial incisional SSI, deep incisional SSI, organ/space SSI, wound disruption, sepsis, septic shock, UTI, ventilator > 48 h, unplanned intubation, pneumonia, venous thrombosis requiring therapy, PE, stroke, unplanned admission to ICU, acute renal failure requiring dialysis, progressive renal insufficiency, cardiac arrest requiring CPR, and myocardial infarction.

### Statistical analysis

Statistical analysis was performed in R (Version 4.4.1, Vienna, Austria). For univariate analyses, Pearson’s Chi-square test or Fisher’s exact test was used for categorical variables as appropriate. Independent two-sample *t* test was used for continuous variables. Categorical variables were reported as frequency and percentage, and continuous variables as mean ± standard deviation (SD). A *p* value of < 0.05 was considered statistically significant.

Multivariate logistic regression was used to analyze 30-day outcomes and adjust for demographic characteristics, comorbidities, and operative variables. Only variables with *p* < 0.05 in the univariate analysis were included in the adjustment. An adjusted odds ratio (AOR) and 95% confidence interval (CI) were reported for each outcome with laparoscopic cases used as the reference group.

## Results

### Patient demographics and operative data

A total of 41,404 cases (14,474 SG; 26,930 RYGB) were included in the analysis. Of the 14,474 revisional SG cases, 12,680 were laparoscopic (87.6%, L-SG) and 1,794 were robotic (12.4%, R-SG). Patients who underwent revisional L-SG were significantly younger (48.5 ± 10.8 vs. 49.1 ± 10.8 years, *p* < 0.05) than patients who underwent R-SG. There were significant differences in ASA class between groups as detailed in Table 1. Revisional L-SG patients also had a lower prevalence of hypertension, diabetes mellitus, OSA, and GERD compared to R-SG patients. For operative variables, L-SG patients were more likely to have a leak test performed (75.6% vs. 68.3%, *p* < 0.001) and a drain placed (16.5% vs. 11.0%, *p* < 0.001). Operative time for revisional L-SG was significantly shorter than R-SG (86.4 ± 45.8 vs. 113.5 ± 51.7 min, *p* < 0.001).

**Table 1:** Patient demographics and operative variables for laparoscopic and robotic revisional sleeve gastrectomy

Demographics	L-SG (*N* = 12,680)	R-SG (*N* = 1794)	*p* value
Age, mean ± SD (years)	48.5 ± 10.8	49.1 ± 10.8	** < 0.05**
Female, *n* (%)	10,633 (83.9)	1497 (83.4)	0.68
ASA, *n* (%)			** < 0.05**
1–2	2997 (23.6)	380 (21.2)	
3	9356 (73.8)	1352 (75.4)	
4–5	327 (2.6)	62 (3.5)	
BMI, mean ± SD (kg/m^2^)	43.6 ± 7.9	43.7 ± 8.0	0.57
Comorbidities, *n* (%)			
Hypertension	5740 (45.3)	885 (49.3)	** < 0.01**
Hyperlipidemia	2818 (22.2)	435 (24.3)	0.06
Dependent functional status	163 (1.3)	14 (0.8)	0.09
Smoking	766 (6.0)	95 (5.3)	0.23
Diabetes mellitus	2182 (17.2)	348 (19.4)	** < 0.05**
Chronic steroid/immunosuppression	291 (2.3)	54 (3.0)	0.08
Chronic obstructive pulmonary disease	130 (1.0)	22 (1.2)	0.51
Pulmonary embolism	226 (1.8)	39 (2.2)	0.29
Deep vein thrombosis requiring therapy	258 (2.0)	35 (2.0)	0.88
Therapeutic anticoagulation	424 (3.3)	61 (3.4)	0.96
Inferior vena cava filter	69 (0.5)	8 (0.5)	0.72
Obstructive sleep apnea	3803 (30.0)	607 (33.8)	** < 0.01**
Gastroesophageal reflux disease	4084 (32.2)	626 (34.9)	** < 0.05**
History of myocardial infarction	159 (1.3)	19 (1.1)	0.56
Previous cardiac surgery	161 (1.3)	20 (1.1)	0.66
Renal insufficiency	61 (0.5)	8 (0.5)	0.98
Dialysis	20 (0.2)	6 (0.3)	0.17
Operative variables			
Leak test, *n* (%)	9589 (75.6)	1226 (68.3)	** < 0.001**
Drain placed, *n* (%)	2086 (16.5)	197 (11.0)	** < 0.001**
Operative time, mean ± SD (minutes)	86.4 ± 45.8	113.5 ± 51.7	** < 0.001**

*L-SG*  Laparoscopic sleeve gastrectomy, *R-SG*  robotic sleeve gastrectomy, *SD*  standard deviation, *ASA*  American Society of Anesthesiologists, *BMI*  body mass index

Bold values indicate statistical significance

Of the 26,930 revisional RYGB cases, 21,755 were laparoscopic (80.8%, L-RYGB) and 5,175 were robotic (19.2%, R-RYGB). Patients who underwent L-RYGB were less likely to be female (88.0% vs. 90.2%, *p* < 0.001) compared to R-RYGB. There were significant differences in ASA class between groups as detailed in Table 2. L-RYGB patients were less likely to have preoperative GERD (58.0% vs. 64.5%, *p* < 0.001) and more likely to have a drain placed during surgery (26.6% vs. 12.8%, *p* < 0.001). Operative time for revisional L-RYGB was significantly shorter than R-RYGB (130.7 ± 64.7 vs. 165.5 ± 66.8 min, *p* < 0.001).

**Table 2:** Patient demographics and operative variables for laparoscopic and robotic revisional Roux-en-Y gastric bypass

Demographics	L-RYGB (*N* = 21,755)	R-RYGB (*N* = 5175)	*p* value
Age, mean ± SD (years)	46.7 ± 10.6	46.7 ± 10.7	0.97
Female, n (%)	19,148 (88.0)	4669 (90.2)	** < 0.001**
ASA, n (%)			** < 0.05**
1–2	5603 (25.8)	1245 (24.1)	
3	15,602 (71.7)	3790 (73.2)	
4–5	550 (2.5)	140 (2.7)	
BMI, mean ± SD (kg/m^2^)	41.5 ± 8.3	41.4 ± 8.2	0.92
Comorbidities, n (%)			
Hypertension	8447 (38.8)	2043 (39.5)	0.40
Hyperlipidemia	4236 (19.5)	1032 (19.9)	0.45
Dependent functional status	158 (0.7)	45 (0.9)	0.33
Smoking	1097 (5.0)	227 (4.4)	0.05
Diabetes mellitus	3383 (15.6)	822 (15.9)	0.57
Chronic steroid/immunosuppression	504 (2.3)	140 (2.7)	0.11
Chronic obstructive pulmonary disease	263 (1.2)	76 (1.5)	0.15
Pulmonary embolism	385 (1.8)	89 (1.7)	0.85
Deep vein thrombosis requiring therapy	503 (2.3)	107 (2.1)	0.31
Therapeutic anticoagulation	652 (3.0)	175 (3.4)	0.16
Inferior vena cava filter	126 (0.6)	19 (0.4)	0.08
Obstructive sleep apnea	6010 (27.6)	1409 (27.2)	0.58
Gastroesophageal reflux disease	12,611 (58.0)	3336 (64.5)	** < 0.001**
History of myocardial infarction	230 (1.1)	53 (1.0)	0.89
Previous cardiac surgery	197 (0.9)	51 (1.0)	0.65
Renal insufficiency	77 (0.4)	27 (0.5)	0.10
Dialysis	34 (0.2)	7 (0.1)	0.88
Operative variables			
Leak test, n (%)	19,598 (90.1)	4693 (90.7)	0.20
Drain placed, n (%)	5776 (26.6)	660 (12.8)	** < 0.001**
Operative time, mean ± SD (minutes)	130.7 ± 64.7	165.5 ± 66.8	** < 0.001**

*L-RYGB*  Laparoscopic Roux-en-Y gastric bypass, *R-RYGB*  robotic Roux-en-Y gastric bypass, *SD*  standard deviation, *ASA*  American Society of Anesthesiologists, *BMI*  body mass index

Bold values indicate statistical significance

### Robot utilization

**Fig. 2:**
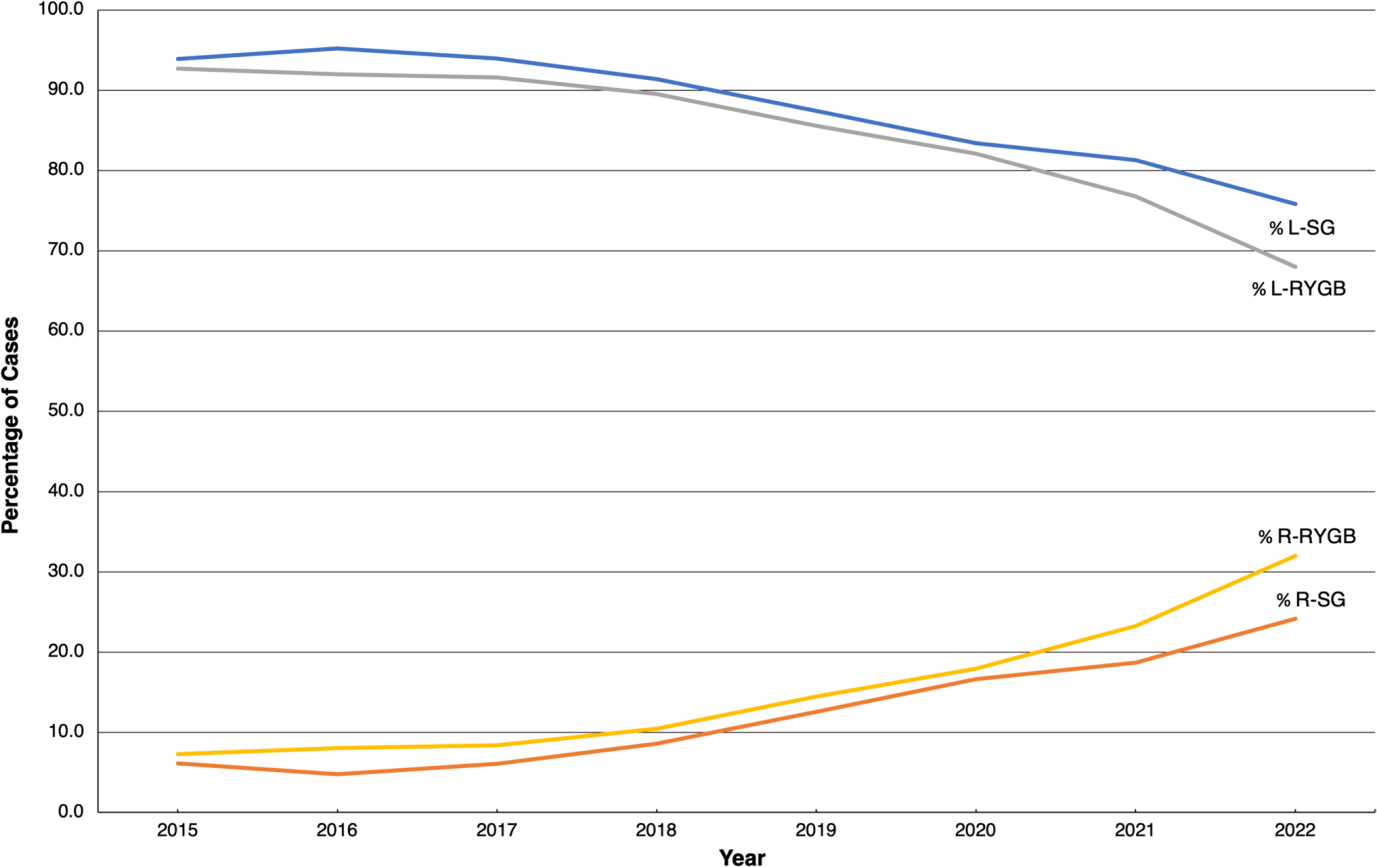
Percentage of revisional sleeve gastrectomy (SG) and Roux-en-Y gastric bypass (RYGB) cases performed laparoscopically (L-) and robotically (R-) from 2015 to 2022

The total number of revisional R-SG cases increased by 5.3 times from 2015 (86 cases) to 2022 (453 cases). In contrast, the total number of revisional L-SG cases increased by 1.08 times from 2015 (1317 cases) to 2022 (1420 cases). The total number of revisional R-RYGB cases increased substantially by 22.4 times from 2015 (89 cases) to 2022 (1995 cases). The number of revisional L-RYGB cases increased by 3.7 times from 2015 (1130) to 2022 (4236). Taken together, the percentage of all revisional SG and RYGB performed robotically increased from 6.1% and 7.3% to 24.2% and 32.0%, respectively, over the 8-year period (Fig. 2).

### Outcomes

Outcomes for revisional SG are detailed in Table 3. Compared to R-SG, L-SG had a significantly lower risk of postoperative pulmonary embolism (0.08% vs. 0.28%, *p* = 0.03, AOR 3.72 [1.25–10.99]). There were otherwise no significant differences between L-SG and R-SG in terms of overall morbidity, leak, bleeding, readmission, reoperation, reintervention, mortality, length of stay, or any other postoperative complications.

**Table 3:** 30-Day outcomes for laparoscopic and robotic revisional sleeve gastrectomy

Outcome, *n* (%)	L-SG (*N* = 12,680)	R-SG (*N* = 1,794)	*p* value	AOR (95% CI)
Overall morbidity	408 (3.2)	69 (3.8)	0.19	1.23 (0.95–1.60)
Aggregate leak Reoperation for leak Intervention for leak Readmission for leak Organ/space SSI Drain present at 30 days* Anastomotic/staple line leak**	131 (1.0) 45 (0.35) 40 (0.32) 61 (0.48) 88 (0.69) 34 (0.41) 26 (0.59)	15 (0.8) 4 (0.22) 3 (0.17) 6 (0.33) 16 (0.89) 5 (0.72) 4 (0.36)	0.51 0.49 0.40 0.50 0.44 0.23 0.49	0.91 (0.53–1.57) 0.67 (0.24–1.87) 0.57 (0.18–1.84) 0.72 (0.31–1.67) 1.35 (0.79–2.32) – –
Aggregate bleeding Reoperation for bleeding Intervention for bleeding Readmission for bleeding GI tract bleeding**	45 (0.35) 29 (0.23) 5 (0.04) 8 (0.06) 18 (0.41)	2 (0.11) 1 (0.06) 0 0 1 (0.09)	0.12 0.17 0.87 0.61 0.15	0.30 (0.07–1.24) 0.24 (0.03–1.78) – – –
Blood transfusion	62 (0.49)	9 (0.50)	0.99	1.05 (0.52–2.12)
Readmission	451 (3.6)	57 (3.2)	0.45	0.89 (0.67–1.17)
Reoperation	189 (1.5)	25 (1.4)	0.83	0.95 (0.62–1.44)
Reintervention	154 (1.2)	22 (1.2)	0.99	1.05 (0.67–1.65)
Mortality	10 (0.08)	0	0.62	–
Superficial incisional SSI	51 (0.4)	11 (0.6)	0.28	1.54 (0.80–2.98)
Deep incisional SSI	9 (0.07)	3 (0.17)	0.18	2.33 (0.63–8.66)
Wound disruption	5 (0.04)	0	0.87	**–**
Sepsis	27 (0.21)	7 (0.39)	0.18	1.77 (0.77–4.07)
Septic shock	9 (0.07)	2 (0.11)	0.64	1.50 (0.32–7.01)
Urinary tract infection	36 (0.3)	9 (0.5)	0.19	1.86 (0.89–3.88)
Ventilator > 48 h	8 (0.06)	3 (0.17)	0.15	2.52 (0.66–9.61)
Unplanned intubation	10 (0.08)	4 (0.22)	0.09	2.58 (0.80–8.28)
Pneumonia	26 (0.21)	7 (0.39)	0.18	1.80 (0.78–4.18)
Venous thrombosis requiring therapy	30 (0.24)	9 (0.50)	0.05	2.20 (1.04–4.67)
Pulmonary embolism	10 (0.08)	5 (0.28)	**0.03**	**3.72 (1.25–10.99)**
Stroke	1 (0.01)	1 (0.06)	0.23	7.55 (0.43–134.26)
Unplanned admission to ICU	78 (0.6)	10 (0.6)	0.89	0.89 (0.46–1.73)
Acute renal failure requiring dialysis	8 (0.06)	0	0.61	–
Progressive renal insufficiency	11 (0.09)	1 (0.06)	0.99	0.57 (0.07–4.47)
Cardiac arrest requiring CPR	7 (0.06)	0	0.99	–
Myocardial infarction	2 (0.02)	0	0.99	–
Length of stay, mean ± SD (days)	1.49 (1.41)	1.49 (1.98)	0.96	–

^*^variable only present in 2015–2019 datasets; **variable only present in 2020–2022 datasets

*L-SG*  Laparoscopic sleeve gastrectomy, *R-SG*  robotic sleeve gastrectomy, *AOR*  adjusted odds ratio, *CI*  confidence interval, *SSI*  surgical site infection, *GI*  gastrointestinal, *ICU*  intensive care unit, *CPR*  cardiopulmonary resuscitation

Bold values indicate statistical significance

Outcomes for revisional RYGB are detailed in Table 4. Compared to R-RYGB, L-RYGB had significantly higher rates of overall morbidity (6.2% vs. 4.8%, *p* < 0.001, AOR 0.80 [0.70–0.93]), blood transfusion (1.5% vs. 1.0%, *p* < 0.05, AOR 0.74 [0.55–0.99]), superficial incisional SSI (1.2% vs. 0.4%, *p* < 0.001, AOR 0.30 [0.19–0.47]), and longer length of stay (1.87 vs. 1.76 days, *p* < 0.001). Conversely, L-RYGB had lower rates of pulmonary complications, including ventilator > 48 h (0.15% vs. 0.29%, *p* < 0.05, AOR 1.98 [1.06–3.70]) and unplanned intubation (0.19% vs. 0.35%, *p* < 0.05, AOR 2.08 [1.18–3.67]). There were otherwise no significant differences between L-RYGB and R-RYGB in terms of leak, bleeding, readmission, reoperation, reintervention, mortality, or any other postoperative complications.

**Table 4:** 30-Day outcomes for laparoscopic and robotic revisional Roux-en-Y gastric bypass

Outcome, n (%)	L-RYGB (N = 21,755)	R-RYGB (N = 5175)	*p* value	AOR (95% CI)
Overall morbidity	1341 (6.2)	246 (4.8)	** < 0.001**	**0.80 (0.70–0.93)**
Aggregate leak Reoperation for leak Intervention for leak Readmission for leak Organ/space SSI Drain present at 30 days* Anastomotic/staple line leak**	262 (1.2) 101 (0.46) 52 (0.24) 71 (0.33) 193 (0.89)	54 (1.0) 25 (0.48) 12 (0.23) 21 (0.41) 49 (0.95)	0.37 0.95 0.99 0.45 0.74	1.00 (0.74–1.35) 1.12 (0.72–1.75) 1.11 (0.59–2.10) 1.38 (0.84–2.26) 1.11 (0.81–1.53) – –
Aggregate bleeding Reoperation for bleeding Intervention for bleeding Readmission for bleeding GI tract bleeding**	207 (0.95) 76 (0.35) 48 (0.22) 73 (0.34)	51 (0.99) 10 (0.19) 7 (0.14) 23 (0.44)	0.88 0.10 0.29 0.29	1.09 (0.80–1.49) 0.61 (0.31–1.19) 0.64 (0.29–1.42) 1.37 (0.85–2.20)
Blood transfusion	329 (1.5)	54 (1.0)	** < 0.05**	**0.74 (0.55–0.99)**
Readmission	1495 (6.9)	386 (7.5)	0.14	1.10 (0.98–1.24)
Reoperation	685 (3.1)	166 (3.2)	0.86	1.07 (0.90–1.27)
Reintervention	532 (2.4)	112 (2.2)	0.25	0.91 (0.74–1.12)
Mortality	23 (0.11)	9 (0.17)	0.29	1.87 (0.85–4.13)
Superficial incisional SSI	270 (1.2)	19 (0.4)	** < 0.001**	**0.30 (0.19–0.47)**
Deep incisional SSI	58 (0.27)	7 (0.14)	0.12	0.54 (0.25–1.20)
Wound disruption	19 (0.09)	3 (0.06)	0.79	0.68 (0.19–2.32)
Sepsis	70 (0.32)	16 (0.31)	0.99	1.01 (0.58–1.75)
Septic shock	35 (0.16)	15 (0.29)	0.08	1.93 (0.98–3.58)
Urinary tract infection	115 (0.53)	19 (0.37)	0.17	0.66 (0.41–1.08)
Ventilator > 48 h	32 (0.15)	15 (0.29)	** < 0.05**	**1.98 (1.06–3.70)**
Unplanned intubation	41 (0.19)	18 (0.35)	** < 0.05**	**2.08 (1.18–3.67)**
Pneumonia	102 (0.47)	27 (0.52)	0.70	1.12 (0.73–1.72)
Venous thrombosis requiring therapy	38 (0.17)	13 (0.25)	0.33	1.49 (0.79–2.82)
Pulmonary embolism	45 (0.21)	10 (0.19)	0.98	1.05 (0.53–2.11)
Stroke	1 (0.005)	1 (0.02)	0.35	5.96 (0.33–108.97)
Unplanned admission to ICU	248 (1.1)	54 (1.0)	0.60	0.97 (0.72–1.30)
Acute renal failure	13 (0.06)	3 (0.06)	0.99	1.16 (0.32–4.12)
Progressive renal insufficiency	12 (0.06)	4 (0.08)	0.53	1.54 (0.48–4.87)
Cardiac arrest requiring CPR	15 (0.07)	5 (0.10)	0.57	1.56 (0.56–4.37)
Myocardial infarction	5 (0.02)	2 (0.04)	0.63	1.35 (0.26–7.03)
Length of stay, mean ± SD (days)	1.87 (2.09)	1.76 (2.17)	** < 0.001**	–

^*^variable only present in 2015–2019 datasets; **variable only present in 2020–2022 datasets

*L-RYGB*  Laparoscopic Roux-en-Y gastric bypass, *R-RYGB*  robotic Roux-en-Y gastric bypass, *AOR*  adjusted odds ratio, *CI*  confidence interval, *SSI*  surgical site infection, *GI*  gastrointestinal, *ICU*  intensive care unit, *CPR*  cardiopulmonary resuscitation

Bold values indicate statistical significance

## Discussion

This study represents the largest retrospective comparison of laparoscopic and robotic revisional SG and RYGB outcomes in the literature, and to our knowledge, the first study reporting these outcomes from the 2022 iteration of the MBSAQIP database. As robot utilization increases across surgical disciplines, it is important to understand the role of robotics in MBS, particularly in revisional cases, as the technical advantages of robotic platforms may be most apparent in these challenging operations. In addition, complication rates for reoperative MBS are generally higher than primary MBS, highlighting the need for technical strategies to optimize patient outcomes [5, 11, 24].

Our study demonstrated a single statistically significant difference in 30-day outcomes between laparoscopic and robotic revisional SG cases, with R-SG having a higher rate of postoperative pulmonary embolism, though absolute rates in both approaches were low. This may be related to longer operative times or the higher rate of comorbidities in the R-SG group. Outcomes between L-SG and R-SG were otherwise equivalent. In contrast, there were several significant differences between laparoscopic and robotic revisional RYGB, with R-RYGB having lower overall morbidity, blood transfusion, superficial incisional SSI, and shorter length of stay. The shorter length of stay for R-RYGB may be a consequence of the lower overall morbidity, though additional factors not captured in this dataset such as postoperative pain may be contributors. R-RYGB did however have a higher risk of ventilator > 48 h and unplanned intubation, which may also be related to the longer operative time and anesthesia duration with robotic cases, though absolute rates of these complications were also low [25]. It may be argued that these respiratory complications present a more significant risk of morbidity to patients than SSI and blood transfusion, and attention to these respiratory complications is warranted in future studies. Overall, despite significantly longer operative times for robotic SG and RYGB, we found robotic revisional MBS to have an acceptable safety profile compared to laparoscopic. In addition, robotic complications for revisional RYGB were overall lower when compared to laparoscopic. The robotic platform may be advantageous in these more complex RYGB cases, as they often require careful and precise dissection of altered anatomy in hostile operative fields. It follows that the technical and ergonomic advantages of robotic platforms are uniquely suited to these procedures.

Several studies comparing laparoscopic and robotic revisional MBS have been performed with similarities and differences to our results. Acevedo et al. performed 1:1 case control-matching of revisional SG and RYGB cases using 2015–2016 MSBAQIP data and similarly found a higher transfusion rate with L-RYGB, while R-SG had a higher rate of postoperative sepsis [19]. Outcomes were otherwise similar between groups in their matched analysis. Nasser et al. analyzed revisional SG and RYGB cases using 2015–2017 MBSAQIP data with multivariate logistic regression and reported R-SG had higher rates of overall morbidity, reintervention, reoperation, ventilator > 48 h, organ space SSI, and sepsis [18]. They also found R-RYGB had a lower rate of pulmonary complications, any SSI, and blood transfusion. In contrast to these reports, we found L-SG and R-SG outcomes to be relatively similar. Smaller cases series, meta-analyses, and propensity-score matched MBSAQIP analyses have also shown no difference in outcomes between laparoscopic and robotic revisional MBS [17, 20–22, 26]. The reasons for the lack of a consistent body of evidence are likely multifactorial. Aggregate progression of surgeons across the robotic MBS learning curve in the newer MBSAQIP data may be a contributor. It is likely that many centers across the country have not yet completed the learning curve for robotic MBS. There may also be shifts in surgeon technique over time not captured in the MBSAQIP that could account for the improved R-SG outcomes in our study. The addition of more granular surgical technique data to the MBSAQIP would be beneficial for future retrospective outcome comparisons. Methodological differences in study design and outcomes reporting are important considerations as well [22]. Due to these factors, analyzing outcomes in robotic surgery is complicated, and randomized controlled trials with long-term follow-up are needed to compare operative approaches in revisional MBS. Nevertheless, the potential for improved outcomes with robotic surgery is promising.

Increasing utilization of robotics in MBS has been demonstrated in multiple studies [13, 27, 28]. A recent study analyzing over 1 million cases from 2015 to 2020 MBSAQIP data found that robotic RYGB cases increased from 6.8% to 16.7%, while robotic SG increased from 6.0% to 17.2% [13]. We found a continued increase in robotic use for revisional MBS through 2022. The widespread adoption of robotics in MBS, and surgery more broadly, remains controversial due to cost concerns with the robotic platform [29, 30]. However, there is evidence that the cost of robotic MBS is decreasing, and single-institution studies have reported no cost difference between the approaches [31–33]. It is well documented that operative times for robotic MBS cases are generally longer than conventional laparoscopy, resulting in longer anesthesia duration for patients [19, 26]. The difference in operative time between laparoscopic and robotic approaches may diminish as surgeons progress across the learning curve. Buchs et al. reported that after overcoming the learning curve for R-RYGB, there was no longer a difference in operative time between laparoscopic and robotic cases [16]. While we identified a clinical benefit of robotics for revisional RYGB despite longer operative times, it remains to be seen how the net cost of robotic platforms evolves with increased utilization nationally.

Determining the optimal surgical approach for revisional MBS will also be critical in the broader context of obesity management. With the recognition of obesity as a chronic, multifactorial disease and the rise of MBS worldwide, the need for revisional surgery is likely to increase. The increasing use of robotic platforms in these complex cases and associated outcomes should be monitored to help patients achieve optimal outcomes and make informed decisions. It is also important to recognize that surgery represents one treatment option in the multidisciplinary approach to obesity management. Pharmacologic therapy in the form of glucagon-like peptide 1 (GLP-1) receptor agonists has emerged as a promising treatment for weight regain [34]. There are also several emerging incretin-based therapies that are likely to influence the obesity management landscape [35]. Counseling patients on the risks and benefits of revisional surgery as part of shared decision-making discussions may be directly influenced by advances in surgical technique and potential patient safety benefits afforded by robotic platforms. The efficacy of revisional surgery will also need to be weighed against the risk of morbidity as medical therapies continue to advance.

There are several limitations to this retrospective study of a large national database. Many important and potentially confounding variables are not available in the MBSAQIP, such as years of surgeon experience with robotic MBS, hospital robotic case volume, anastomosis techniques (handsewn, stapled, or combination), and whether cases were totally or partially robotic. Before 2019, a robotic stapler was not widely available, and consequently, all stapling was performed laparoscopically by a bedside assistant. It is unclear how surgeon practices have changed with the introduction of robotic staplers and how these changes may have impacted patient outcomes. For 2015–2019 data, it is not possible to know what the index operation was that required a revision/conversion, which may directly influence case complexity, complications, and operative time. Technical details regarding the type of revisional surgery performed are also missing from current data. Clinical criteria for the choice of operative approach are not available, which may introduce selection bias in a surgeon’s preference to perform cases robotically. The MBSAQIP also only provides 30-day outcomes data, limiting the ability to assess long-term outcomes.

## Conclusion

Robotic and laparoscopic revisional MBS are both safe and effective surgical approaches. Robot utilization in revisional bariatric surgery is increasing annually. Robotic surgery has lower postoperative morbidity and shorter length of stay in revisional RYGB when compared to laparoscopic. Robotic platforms may have the capacity to improve the delivery of care for patients undergoing revisional bariatric surgery.
